# The death risk factors of patients undergoing re-exploration for bleeding or tamponade after isolated off-pump coronary artery bypass grafting: a case–control study

**DOI:** 10.1186/s12872-021-02017-2

**Published:** 2021-04-22

**Authors:** Tongxiao Luan, Yingzhu Zhuang, Weihong Nie, Sumin Yang, Yuhui Wu, Rongmei Wang, Yunyan Dai, Hong Zhang

**Affiliations:** 1 Department of Cardiovascular Surgery, Affiliated Hospital of Qingdao University, 16 Jiangsu Road, Qingdao, 266000 Shandong Province China; 2 Qingdao University, 308 Ningxia Road, Qingdao, 266071 Shandong Province China; 3 Qingdao Fuwai Cardiovascular Hospital, 201 Nanjing Road, Qingdao, 266034 Shandong Province China

**Keywords:** Cardiac tamponade, Bleeding, Re-exploration, Death risk factor, Isolated OPCABG

## Abstract

**Background:**

The purpose of the study is to identify off-pump patients who are at higher risk of mortality after re-exploration for bleeding or tamponade.

**Methods:**

We analyzed the data of 3256 consecutive patients undergoing isolated off-pump coronary artery bypass grafting (OPCABG) in our heart center from 2013 through 2020. Fifty-eight patients underwent re-exploration after OPCABG. The 58 patients were divided into death group and survival group according to their discharge status. Propensity score matching (PSM) was performed to analysis the risk factors of death. 15 pairs of cases of two groups were matched well.

**Results:**

The mortality rate of patients underwent re-exploration after OPCABG for bleeding or tamponade was 27.59% (16/58). In the raw data, we found the patients in death group had higher body mass index (BMI) (*P* = 0.030), higher cardiac troponin T (cTnT) (*P* = 0.028) and higher incidence of heart failure before OPCABG (*P* = 0.003). After PSM, the levels of lactic acid before and after re-exploration (*P* = 0.028 and *P* < 0.001) were higher in death group. And the levels of creatinine (*P* = 0.002) and cTnT (*P* = 0.017) were higher in the death group after re-exploration. The death group had longer reoperation time (*P* = 0.010). In addition, the perioperative utilization rate of intra-aortic ballon pump (IABP) (*P* = 0.027), continuous renal replacement therapy (CRRT) (*P* < 0.001) and platelet transfusion (*P* = 0.017) were higher than survival group.

**Conclusions:**

The mortality rate of patients undergoing re-exploration for bleeding or tamponade after isolated OPCABG is high. More attention should be paid to patients with above risk factors and appropriate measures should be taken in time.

## Background

At present, cardiovascular disease is the leading global cause of death. About 17.8 million people died of cardiovascular disease every year [1]. Coronary heart disease (CHD) ranks first in cardiac death (47.8% of all cardiovascular deaths) [2], which has become the first medical burden [3]. Coronary artery bypass grafting (CABG), including OPCABG and on-pump coronary artery bypass grafting (ONCABG), is one of the main methods for the treatment of CHD. OPCABG can avoid or reduce potential side effects of cardiopulmonary bypass, such as systemic inflammatory response, neurocognitive decline, renal dysfunction, pulmonary dysfunction, gaseous microemboli, and multiple organ failure [4]. Furthermore, OPCABG has more advantages than ONCABG in the patients with previous CABG, diabetics, left ventricular ejection fraction (LVEF) between 30 and 50%, females, or age 66–75 years [5]. So OPCABG has become the predominant approach to revascularization at our institution. Postoperative hemorrhage or cardiac tamponade can cause cardiac arrest, cardiogenic shock and other serious adverse events, so re-exploration is usually required to perform to avoid further deterioration. However, re-exploration for bleeding or tamponade is a lethal complication of cardiac surgery, with a detrimental effect that surpasses that of any other known risk factor [6]. The mortality rate for OPCABG patients undergone re-exploration after cardiac surgery was 9–26% [6–8]. Our data showed that the mortality rate of patients undergone re-exploration for bleeding or tamponade was 27.6%. But there is no study to analyze the death factors of patients with re-exploration for bleeding or tamponade after isolated OPCABG. To clarify the death risk factors of these patients and to provide guidance for clinical work, we retrospectively analyzed the prognosis of patients with re-exploration for bleeding or tamponade after isolated OPCABG in our cardiac center.

## Methods

### Objects

**Fig. 1:**
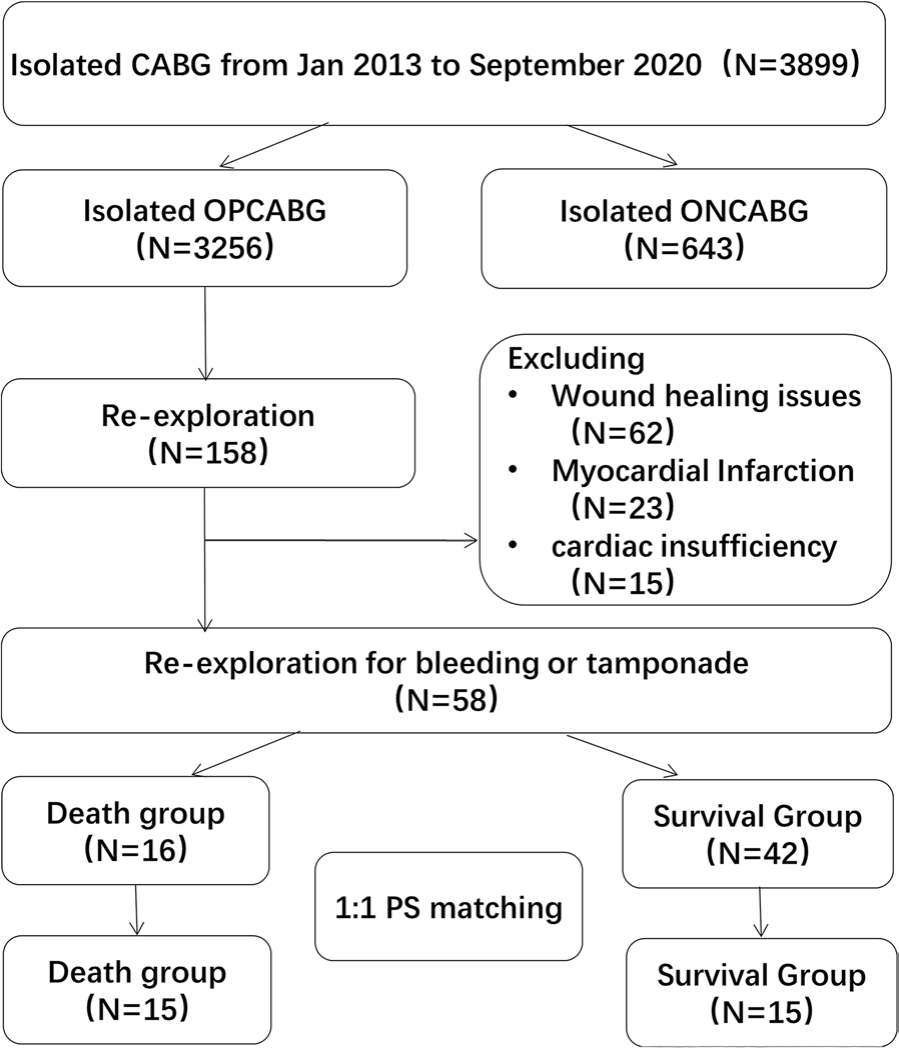
Flow chart of patients screened for inclusion in the study

This was a retrospective observational study. A total of 3256 consecutive patients undergoing isolated OPCABG in the Affiliated Hospital of Qingdao University during Jan 2013 to September 2020 were selected, and finally 58 patients were included in this study. The patients were divided into two groups: death group (n = 16) and survival group (n = 42). PSM analyses was performed to analysis the risk factors of death. Patients were matched into 2 groups of 15 patients each for further analysis. The end points of the study were in-hospital death or successful discharge. Flow chart of patients screened for inclusion in this study are presented in Fig. 1.

### Enrollment

Inclusion criteria: (1) isolated OPCABG; (2) unplanned re-exploration for bleeding or tamponade. Exclusion criteria: (1) ONCABG; (2) hybrid operation; (3) re-exploration due to early postoperative myocardial infarction or cardiac insufficiency; (4) re-exploration due to poor wound healing. The criteria for unplanned re-exploration for bleeding or tamponade are as follows: (1) the drainage volume is greater than that of 200 ml/h for 3 h (2) sudden massive bleeding; (3) cardiac tamponade is indicated by echocardiography; (4) hemodynamic disorders. The study protocol was conducted by the Declaration of Helsinki and was approved by Ethics Committee of Affiliated Hospital of Qingdao University (QYFYWZLL 26117-02).

### Clinical and laboratory measurements

The general data and preoperative information of the patients are shown in Table 1. We analyzed the changes of perioperative biochemical indexes (cTnT, alanine transaminase, creatinine, lactic acid, oxygen partial pressure (PO_2_) and PCO_2_, etc.) (Table 2). Cardiac echocardiography was used to evaluate the changes of cardiac function. The time of reoperation were recorded. The utilization rates of IABP and CRRT, and blood transfusion (plasma, red blood cell, platelet and cryoprecipitate) were analyzed (Table 3). Finally, the direct cause of death was counted (Table 4).

**Table 1:** Preoperative patient characteristics

	Overall patients			Matched population		
	Survival group (N = 42)	Death group (N = 16)	*P* value	Survival group (N = 15)	Death group (N = 15)	*P* value
Sex, male	36(85.71%)	12(75%)	0.564	14(93.33%)	11(73.33%)	0.330
Age, years	64.67 ± 8.02	63.75 ± 10.10	0.719	63.13 ± 6.90	63.533 ± 10.41	0.902
BMI, kg/m^2^	24.02 ± 3.23	26.24 ± 3.52^A^	0.030	24.53 ± 3.22	26.24 ± 3.52	0.062
Hypertension	25(59.52%)	11(68.75%)	0.517	6(40%)	11(73.33%)	0.139
Diabetes	17(40.48%)	7(43.75%)	0.821	4(26.67%)	6(40%)	0.700
Carotid artery stenosis	17(47.22%)^D^	9(69.23%)^C^	0.173	8(53.33%)	9(69.23%)^B^	0.560
Cerebral infarction	8(19.05%)	5(31.25%)	0.520	4(26.67%)	5(33.33%)	1.000
Heart insufficiency	10(23.81%)	10(66.67%)^A^	0.003	4(26.67%)	10(66.67%)	0.066
Respiratory insufficiency	17(47.22%)^D^	6(42.86%)^B^	0.781	4(26.67%)	5(33.33%)^B^	1.000
Hepatic insufficiency	11(27.5%)^B^	4(26.67%)^A^	1.000	4(26.67%)	4(26.67%)	1.000
Renal insufficiency	7(17.5%)^B^	2(13.33%)^A^	0.895	0(0%)	2(13.33%)	0.483
Time between myocardial infarction and surgery, days, (Q1–Q3)	14(10–16)	11.50(7.25–17.75)	0.239	15.67 ± 7.05	13.6 ± 7.64	0.239

BMI, body mass index; carotid artery stenosis was diagnosed by Doppler ultrasonography; Heart insufficiency, LVEF < 50%; Respiratory insufficiency, PO_2_ < 83 mmHg; Hepatic insufficiency, ALT > 50 U/L; Renal insufficiency, creatinine > 132μmoI/L; Missing data: ^A^out of 1 patient; ^B^out of 2 patients; ^C^out of 3 patients; ^D^out of 6 patients

**Table 2:** Biochemical criterion during hospitalization

	Overall patients			Matched population		
	Survival group (N = 42)	Death group (N = 16)	*P* value	Survival group (N = 15)	Death group (N = 15)	*P* value
*Before OPCABG*						
cTnT, ug/L	0.019(0.02)	0.073(0.68)	0.028	0.016(0.02)	0.079(0.61)	0.110
PCO_2_, mmHG	39.14 ± 4.71	38.41 ± 5.70	0.648	39.10 ± 3.98	38.84 ± 5.05	0.878
PO_2_, mmHG	83(51)	96.4(55)	0.804	84.2(50)	96.85(53)	0.803
Lactic acid, mmol/L	1.48 ± 0.62	1.38 ± 0.78	0.621	1.37 ± 0.51	1.23 ± 0.59	0.477
*Before re-exploration*						
ALT, U/L	31(30.5)	21(25)	0.432	30(23.48)	23(29)	0.604
Creatinine, μmoI/L	89(46.5)	97.5(56.5)	0.170	96(38.2)	98(50)	0.494
cTnT, ug/L	0.11(2.04)^C^	0.54(7.42)^A^	0.069	0.279(1.77)^A^	0.539(8.19)^A^	0.062
PCO_2_, mmHG	47.39 ± 11.97	44.16 ± 10.08	0.343	45.95 ± 12.42	44.57 ± 10.29	0.743
PO_2_, mmHG	67.9(39.9)	59.35(12.13)	0.689	53.22 ± 30.98	50.9 ± 14.20	0.821
Lactic acid, mmol/L	3(5.75)	9.2(11.93)	0.011	5.05 ± 3.00	8.38 ± 7.69	0.028
*After re-exploration*						
ALT,U/L	89(391)	554(3717)	0.063	85(306)	582(3656)	0.052
Creatinine, μmoI/L	124(117)	217.5(161)	0.008	119.87 ± 94.15	233.59 ± 118.56	0.002
cTnT, ug/L	0.17(1.92)^B^	2.44(7.79)^A^	0.124	0.407(0.83)	3.457(5.59)^A^	0.017
PCO_2_, mmHG	43.3(15.6)	45.55(22.55)	0.021	42(13.7)	47(24)	0.101
PO_2_, mmHG	54.8(21.05)	59.7(34.87)	0.465	65.6(14.2)	63.1(36.90)	0.724
Lactic acid, mmol/L	2.9(2.5)	10.45(14.38)	< 0.001	2.6(1.9)	11.4(11.8)	< 0.001

cTnT, cardiac troponin T; PCO_2_, partial pressure of carbon dioxide; PO_2_, partial pressure of oxygen; ALT, alanine transaminase; The index is the maximum value in 24 h; Missing data: ^A^out of 3 patients; ^B^out of 4 patients; ^C^out of 8 patients; The “after-exploration” values were taken the peak value within 24 h after return re-exploration

**Table 3:** Other indicators during hospitalization

	Overall patients			Matched population		
	Survival group (N = 42)	Death group (N = 16)	*P* value	Survival group (N = 15)	Death group (N = 15)	*P* value
IABP	18(42.86%)	12(75%)	0.029	4(26.67%)	11(73.33%)	0.027
CRRT	5(11.90%)	9(56.25%)	< 0.001	0(0%)	9(60%)	< 0.001
FFP, ml	3260(3290)	13,552(25,450)	0.050	3260(3120)	11,600(21,790)	0.071
Red blood cell, unit	8(5.5)	14(19.75)	0.201	7.8(4.5)	13.5(24)	0.194
Platelet transfusion	4(9.52%)	7(43.75%)	0.009	0(0%)	6(40%)	0.017
Cryoprecipitate transfusion	17(40.48%)	6(37.5%)	0.836	4(26.67%)	6(40%)	0.700
Time of re-exploration, min	130(45)	225(64)	0.002	148.73 ± 30.93	212.47 ± 84	0.010

Drain output includes all the drainage fluid; IABP, intra-aortic ballon pump; CRRT, continuous renal replacement therapy; FFP, fresh frozen plasma; 1 unit Red blood cell means red blood cells extracted from 200 ml of blood

**Table 4:** Deaths detailing

	Death cases (N = 16)	Incidence
*The reason for re-exploration*		
Tamponade	14	87.5%
Bleeding	2	12.5%
Time between OPCABG and re-exploration, days, (Q1–Q3)	3(2–8.75)	-
*IABP*		
Yes	12	75%
No	4	25%
*CRRT*		
Yes	9	56.25%
No	7	43.75%
Time between re-exploration and death, days, (Q1–Q3)	17(3–22.75)	–
*Direct cause of death*		
Low cardiac output syndrome	8	50%
Heart arrest	4	25%
Malignant ventricular arrhythmia	3	18.75%
Multiple organ failure	3	18.75%
Infectious shock caused by pulmonary infection	4	25%
DIC caused by infectious shock	2	12.5%
Cerebral complications	1	6.25%

DIC, disseminated intravascular coagulation; All patients refused autopsy; IABP, intra-aortic ballon pump; CRRT, continuous renal replacement therapy

### Statistical analysis

SPSS 26.0 was used for data analysis (SPSS Inc., Chicago, Illinois, United States). Kolmogorov–Smirnov Goodness was used to test the normality of quantitative data. The quantitative data of normal distribution were expressed by ${\overline{\text{x}}} \pm {\text{s}}$ and the t-test was used for those groups. The quantitative data of non-normal distribution were expressed by M (IQR), and the comparison between groups was performed by nonparametric Mann–Whitney U test. Classified data are expressed in terms of frequency and percentage. Chi-squared test, corrected chi-squared test or Fisher's exact probability test were used to compare between groups. Propensity matching was done in a 1:1 ratio with a control patient using the nearest-neighbor matching and the following variables were selected: sex, age, BMI, hypertension, diabetes, carotid artery stenosis, cerebral infarction, heart insufficiency, respiratory insufficiency, hepatic insufficiency, renal insufficiency, time between myocardial infarction and surgery (Table 1), and cTnT, PCO_2_, PO_2_, lactic acid before surgery (Table 2). Outcomes are compared in the matched population using t-test, Mann–Whitney U test and Fisher's exact probability test. *P* < 0.05 was statistically significant.

## Results

In this study, the rate of re-exploration for bleeding or tamponade after isolated OPCABG was 1.78%, and the mortality rate was 27.6%. In the raw data, compared with survival group, there were higher BMI (24.02 ± 3.23 kg/m^2^, vs. 26.24 ± 3.52 kg/m^2^, *P* = 0.030), higher ratio of heart insufficiency (23.81% vs. 66.67%, *P* = 0.003), and higher cTnT (0.019 ug/L, vs. 0.073 ug/L, *P* = 0.028) in the death group before OPCABG (Table 1). After PSM, the levels of lactic acid before and after re-exploration (5.05 ± 3.00 mmol/L, vs. 8.38 ± 7.69 mmol/L, *P* = 0.028, and 2.6 mmol/L, vs. 11.4 mmol/L, *P* < 0.001) were higher in death group. And the levels of creatinine (119.87 ± 94.15moI/L, vs. 233.59 ± 118.56moI/L, *P* = 0.002) and cTnT (0.407ug/L, vs. 3.457 ug/L, *P* = 0.017) were higher in the death group after re-exploration. The death group had longer reoperation time (148.73 ± 30.93 min, vs. 212.47 ± 84 min, *P* = 0.010). In addition, the perioperative utilization rates of IABP (26.67% vs. 73.33%, *P* = 0.027), CRRT (0% vs. 60%, *P* < 0.001) and platelet transfusion (0% vs. 40%, *P* < 0.017) were higher in the death group (Tables 2, 3). More details of the 16 patients in death group were shown in Table 4.

## Discussion

OPCABG is most commonly used method permitting surgical intervention to manage CHD at our institution. Postoperative hemorrhage or tamponade is a serious surgical complication. In this study, the mortality rate of patients undergone re-exploration for bleeding or tamponade was as high as 27.6%. The reasons are not only related to early postoperative anticoagulation and antiplatelet therapy, but also related to surgical techniques, so careful hemostasis is the most effective measure to reduce the risk of bleeding and tamponade. Re-exploration often brings secondary injury to patients, even life-threatening. Therefore, it is of great significance to analyze the high risk factors of death in those patients, which can provide necessary guidance for clinical treatment.

In this study, before PSM, there were no statistically significant differences in age, sex, hypertension, diabetes mellitus or cerebral infarction, but BMI between the two groups. The study showed that high BMI was a significant independent predictor for adverse outcomes and prolonged hospitalization after CABG [9]. It might affect the heart through a variety of risk factors, such as dyslipidemia, hypertension, glucose intolerance, inflammatory markers, obstructive sleep apnea/hypoventilation, the prethrombotic state and other unknown mechanisms [10]. The possible direct effect of high BMI on the heart was the heavy cardiac load, which impact the mortality of patients after reoperation.

The level of myocardial enzymes is correlated with the severity and the timing of myocardial infarction. Increased preoperative myocardial enzyme is an independent risk factor for postoperative cardiac death [11, 12]. In this study, there was no difference in the time from myocardial infarction to operation between the two groups. But the higher level of cTnT after re-exploration is associated with higher mortality rate. The lower preoperative EF before PSM is related to the higher mortality. Wrobel et al. found that the mature surgical risk scores have identified EF as a powerful predictor of perioperative mortality [13]. IABP can effectively provide life support to patients, especially for CHD patients. However, the use of IABP also indicates the patients with severe heart failure. Our study showed that the utilization rate of IABP was higher in the death group.

Renal injury is a common yet potentially serious complication after cardiac surgery and its pathophysiology is complex and multifactorial, which includes several factors such as exogenous and endogenous toxins, metabolic factors, ischemia–reperfusion injury, neurohormonal activation, embolization, hemodynamic alterations, along with inflammation and oxidative stress [14]. On the one hand, due to worse cardiac function or hypotension during the reoperation, the kidneys may suffer from ischemic damage. On the other hand, renal insufficiency may affect the regulation of water, salt, electrolytes and metabolites decreases, especially the function of heart, forming a vicious circle. We found that patients with renal insufficiency had higher mortality and higher perioperative utilization rate of CRRT.

Respiratory failure after cardiac surgery increases hospital mortality, with a probability of about 5–20% [15]. Filsoufi et al. have shown that respiratory failure together with a significant co-disease increases hospital mortality [16]. Our results show that high PCO_2_ was associated with mortality before PSM. While the difference was not statistically significant (42 mmHg, vs. 47 mmHg, *P* = 0.101) after PSM, which may be caused by small sample size. Reoperation often requires secondary intubation, so the risk of lung infection is significantly higher. We found that septic shock caused by pulmonary infection accounted for 25% of all deaths after reoperation. Therefore, for patients after reoperation, respiratory tract care should be strengthened, bacterial culture should be carried out in time, and antibiotics should be used timely and reasonably.

Lactic acid is a marker of anaerobic metabolism, indicating the imbalance between tissue oxygen supply and consumption. Lactic acid is also a sensitive index for the evaluation of microcirculatory disorders [17]. Kristensen et al. [18] in a study of 16,376 patients undergoing cardiac surgery, found that lactic acid is an independent risk factor for early cardiac death after cardiac surgery. We found there was a statistical difference in lactic acid between the survival group and the death group before and after re-exploration. Cardiac tamponade and re-exploration will decrease the perfusion of various organs and tissues, which induce the increase of lactic acid. Hyper lactataemia can lead to myocardial intracellular acidosis, metabolic disorder, and then cause the myocardial cell structure and dysfunction, resulting in a poor prognosis [17]. Therefore, we should pay attention to patient's conditions evens light increase of lactic acid after cardiac surgery, especially after re-exploration.

Blood product transfusion is not only an independent predictor of bleeding-related re-exploration [19], but also associated with postoperative mortality [6]. The results of this study indicate that platelet transfusion is associated with the mortality of re-exploration for bleeding or tamponade after isolated OPCABG, but not with plasma, red blood cells, or cryoprecipitate. Platelet transfusion is associated with uncontrollable bleeding, which predicts poor prognosis. Platelets transfusion may enhance the risk of perioperative myocardial infarction and affect the short-term patency rate of grafts. It suggests that we should be cautious in the use of platelets after operation.

Our results show that the longer reoperation time is associated with the higher mortality. We found it was difficult to clear blood clots and find bleeding points during reoperation. It may induce hypotension and vital organs ischemic damage.

## Limitations

Because the study is a case–control study from a single center, the limitations of the present study includes unavoidable confounding factors and the risk of selection bias. In addition, the small sample size limits the applicability and the use of statistical approaches of this study, such as logistic regression.

## Conclusions

The mortality rate of re-exploration for bleeding or tamponade after isolated OPCABG is high. The differences of lactic acid before and after re-exploration, cTnT and creatinine after re-exploration, reoperation time, the use of IABP or CRRT and platelet transfusion were significant between death and survival groups. It suggests that when abnormalities occur in the above indicators, the treatment plan should be adjusted in time to improve prognosis.

## Data Availability

The datasets used and/or analysed during the current study are available from the corresponding author on reasonable request.

## Abbreviations

OPCABG: Off-pump coronary artery bypass grafting
PSM: Propensity score matching
BMI: Body mass index
cTnT: Cardiac troponin T
PCO_2_: Partial pressure of carbon dioxide
IABP: Intra-aortic ballon pump
CRRT: Continuous renal replacement therapy
CHD: Coronary heart disease
CABG: Coronary artery bypass grafting
ONCABG: On-pump coronary artery bypass grafting
LVEF: Left ventricular ejection fraction
PO_2_: Oxygen partial pressure
EF: Ejection fraction
DIC: Disseminated intravascular coagulation
